# Delivery of DNA-Based Therapeutics for Treatment of Chronic Diseases

**DOI:** 10.3390/pharmaceutics16040535

**Published:** 2024-04-13

**Authors:** Carleigh Sussman, Rachel A. Liberatore, Marek M. Drozdz

**Affiliations:** RenBio Inc., Long Island City, New York, NY 11101, USA

**Keywords:** gene therapy, DNA medicine, adeno-associated virus, AAV, plasmid DNA, electroporation, chronic disease

## Abstract

Gene therapy and its role in the medical field have evolved drastically in recent decades. Studies aim to define DNA-based medicine as well as encourage innovation and the further development of novel approaches. Gene therapy has been established as an alternative approach to treat a variety of diseases. Its range of mechanistic applicability is wide; gene therapy has the capacity to address the symptoms of disease, the body’s ability to fight disease, and in some cases has the ability to cure disease, making it a more attractive intervention than some traditional approaches to treatment (i.e., medicine and surgery). Such versatility also suggests gene therapy has the potential to address a greater number of indications than conventional treatments. Many DNA-based therapies have shown promise in clinical trials, and several have been approved for use in humans. Whereas current treatment regimens for chronic disease often require frequent dosing, DNA-based therapies can produce robust and durable expression of therapeutic genes with fewer treatments. This benefit encourages the application of DNA-based gene therapy to manage chronic diseases, an area where improving efficiency of current treatments is urgent. Here, we provide an overview of two DNA-based gene therapies as well as their delivery methods: adeno associated virus (AAV)-based gene therapy and plasmid DNA (pDNA)-based gene therapy. We will focus on how these therapies have already been utilized to improve treatment of chronic disease, as well as how current literature supports the expansion of these therapies to treat additional chronic indications in the future.

## 1. Introduction

Traditional approaches of medicine and surgery have helped to treat many patients; however, there is a wide scope of indications for which conventional treatment is either inefficient or burdensome to the patient. Such indications, often associated with an underlying genetic cause, require multiple rounds of intervention over an extended period of time, making them chronic by nature. Current prescribed treatments for such indications might modulate the course of the disease or improve symptoms, but they are typically lifelong and have very involved dosing regimens. DNA-based medicines have the potential to make the treatment of such chronic conditions more manageable.

For decades, gene therapy has been an attractive approach to overcome diseases with a known underlying genetic cause [1]. The aim of gene therapy is to manipulate or modify the body’s gene expression for therapeutic benefit [1]. Approaches to gene therapy typically involve inactivating a disease-causing gene, replacing a damaged gene with a healthy copy, or introducing a new or modified gene to combat the disease [1]. Impressively, benefits provided by gene therapy can include complete curing from a disease [1]. Applications of gene therapy can be beneficial when tackling and managing chronic indications that possess multiple underlying causes. 

Historically, a variety of DNA-based products have been established [1,2,3,4]. Such products include, but are not limited to, viral vectors [5,6,7,8,9,10] and pDNA [11,12]. In the case of viral vectors, viruses are modified to eliminate pathogenic attributes, typically including any ability to replicate, then are used as a vehicle to deliver therapeutic genes into cells [5,8]. Similarly, pDNA can be designed to carry therapeutic genes, but pDNA requires an additional delivery component to efficiently enter cells [11]. In both cases, these therapies utilize the host cell as the “factory” to produce the therapeutic protein [2,3,13]. Not only is the format of the therapeutic gene important (viral vectors vs. plasmids), but the delivery method has also been shown to impact efficiency of the approach [14,15,16,17]. While viral vectors are effective in introducing the transgene into a target cell with basic injection [8], pDNA requires an additional delivery method to facilitate efficient uptake by the target cells [14,15,16,17]. Electroporation (EP) is a delivery technique that has been investigated for this purpose and has shown potential when it comes to improving the therapeutic benefit of pDNA treatments (pDNA/EP) [14,15] (Table 1). 

Here, we will review the recent advances in the treatment of chronic diseases with DNA-based medicines with a focus on adeno-associated virus (AAV) and pDNA-based therapies. We will discuss the benefits and limitations of both, highlight the potential of EP for delivery of pDNA-based therapies, and elaborate on how the field may shape in the near future.

## 2. Adeno-Associated Virus-Based Gene Therapy

### 2.1. Attributes of Adeno-Associated Virus-Based Gene Therapy

AAV was discovered in the 1960s as a contaminant in a preparation of simian adenovirus and is defined as a non-enveloped, replication-defective member of the Parvoviridae virus family [5]. AAVs have small icosahedral capsids containing DNA and require co-infection with a helper virus for lytic growth [18,19], and thus pathogenicity [19]. Lower levels of pathogenicity when compared to other viruses [19], along with additional advantages such as the ability to transduce both dividing and non-dividing cells [20,21], make AAV an attractive candidate for gene delivery. The first AAV proof-of-concept study was published in 1984 [22], in which Hermonat and Muzyczka employed AAV to deliver a neomycin resistance gene to mammalian cells in culture [22]. In the decades following, AAV continued to be evaluated for its ability to deliver genes to cells and has made significant strides in reaching important milestones in the clinic [23,24]. Advances culminated in the approval of the first AAV therapy, Glybera, by the European Medicines Agency in 2012 [25] and subsequently by the US Food and Drug Administration in 2017. Although this AAV therapy has recently been discontinued, its withdrawal was due to the cost of treatment rather than any ineffectiveness of the therapy itself [26]. 

The AAV viral genome of ~4.8 kb is composed of linear single-stranded DNA with inverted terminal repeats (ITRs) at both ends [20,27]. The viral genetic material between the ITRs can be replaced with therapeutic genetic material [8]. While an attractive vector, the packaging capacity of ~4.8 kb is a limiting factor [8], making vector design involving larger genes more difficult, and sometimes impossible. Exceeding packaging capacity can have negative effects on the therapy. Repercussions include lower expression efficiency [28,29], and in severe cases, truncation of the transgene, thereby completely abolishing efficacy of the therapy [28]. Subceeding packaging capacity has also shown similar negative effects of reducing expression efficiency [29]. Another consideration when designing an efficient form of AAV gene therapy is the selection of a suitable AAV serotype. There are 13 natural AAV serotypes that are more commonly reported on, with that number likely to grow [18,30]. In addition to the 13 natural serotypes, novel hybrid AAV vectors have been developed and are still being developed [30,31]. Each serotype, regardless of natural or novel identity, has specific receptor and tissue tropisms, as reviewed by Issa et al. [27] (Table 2), with engineered serotypes being generated to modulate such tropisms [32,33,34]. With this in mind, serotype selection is critical for maximizing efficiency of the therapy [18,30,35].

The capacity to select from an array of serotypes for therapy design can be advantageous [30,35], but the ubiquitous nature of AAV infections has the potential to negate the benefits. Since AAVs are endemic and non-pathogenic [19], many individuals do not realize they have been exposed to AAVs. Globally, studies have seen the highest level of serotype positivity (seroprevalence or seropositivity) being associated with AAV2, and lowest associated with AAV5 [36,37]. Exposure to AAVs can correlate with pre-existing immunity to various serotypes, limiting success of treatments [36,38,39]. Testing for pre-existing antibodies against AAVs has been introduced as a strategy to determine treatment eligibility to avoid such issues [38,39,40]. By similar biological mechanisms, repeated exposure to the same AAV therapy can result in immunity to treatment, even if no pre-existing seropositivity was detected [41,42,43,44,45]. The body’s inevitable immune response to AAV treatment, regardless of pre-existing seropositivity, makes repeat treatments with AAV gene therapy challenging [42,43]. A meta-analysis of more than 200 AAV clinical trials found that almost 50% of trials administered immunosuppressants in conjunction with AAV delivery to avoid immune response to treatment [46]. With immunity proven to play a fundamental role in efficiency of treatment, methods to improve AAV technology to avoid such responses are currently being investigated [47,48]. Strategies addressing immunity, as well as other limitations of AAV, include, but are not limited to, optimizing dosing regimen [49], further engineering of AAV capsids [50,51], and vector development such as self-complementary AAVs (scAAVs) [51,52,53,54]. 

### 2.2. AAV-Based Therapy in the Context of Chronic Disease

While there is clearly room for improvement [55,56], AAV still harbors many advantages that make it an attractive treatment for various indications, especially for targeting monogenic recessive diseases [8,23]. 

Hemophilia is one of the many chronic indications that has become a target for AAV-based gene therapy [57,58,59,60]. Hemophilia is a rare inherited disorder in which one’s blood does not clot properly due to insufficient clotting factors [61,62]. There are two types of hemophilia. Hemophilia A is classified as a lack or total absence of clotting factor 8 (FVIII) [61,63], and hemophilia B is classified as a shortage or absence of clotting factor 9 (FIX) [63,64]. The standard of care for hemophilia includes prophylactic intravenous (IV) injections of the missing factor, or injection of antibodies that bridge the biological pathway necessary to restore the function of the clotting signaling cascade [61,65]. Management of hemophilia requires frequent treatments, often requiring administration multiple times per week [61]. In some cases, additional doses must be given following blunt injuries to prevent uncontrolled bleeding [61]. Such frequent administration is costly and time consuming, making it difficult for the patient to adhere to treatment regimens [66]. Therefore, a gene therapy for hemophilia, in which the necessary clotting factors would be continuously produced in the patient’s body, is an attractive alternative [60]. 

With one AAV therapy addressing hemophilia A already approved for use in humans [67], and others in the clinic (ClinicalTrials.Gov; NCT03432520), there is enormous excitement surrounding AAV-based hemophilia treatments [68]. For treatment of hemophilia A, Spark Therapeutics tested its therapy using AAV serotype 3 [60], SPK-8011 (Dirloctocogene Samoparvovec), through an open label phase 1/2 trial (ClinicalTrials.Gov; NCT03003533) as well as a long-term study (ClinicalTrials.Gov; NCT03432520). These studies confirmed the safety profile of the therapy and also evaluated the expression pattern of FVIII in the years following treatment [69]. A single IV infusion of Dirloctocogene Samoparvovec was jointly administered with glucocorticoids [69] and resulted in sustained FVIII expression in 16 of 18 participants. Expression of FVIII lasted more than one year post-therapy, and in many, was stably maintained for more than two years [69]. These data highlight the tremendous benefits that AAV therapy can provide over standard treatments; compared to standard treatments for hemophilia, AAV requires fewer interventions to yield the same therapeutic effects. Of the 18 participants, the two that did not respond to the therapy experienced an immune response to the AAV, despite the administration of glucocorticoids [69]. 

Spark Therapeautics has also made strides with another AAV gene therapy, SPK-9001 (Fidanacogene Elaparvovec), for hemophilia B [70,71]. This AAV therapy, utilizing recombinant AAV serotype rh74 [72], has advanced through similar phase 1/2 trials (ClinicalTrials.Gov; NCT02484092, ClinicalTrials.Gov; NCT03307980). Data obtained showed that one-time IV infusion of Fidanacogene Elaparvovec almost completely eliminated the need for exogenous FIX treatment, with the follow-up being one year after administration [70]. Based on the success of earlier trials, a phase 3 trial is ongoing to track long-term expression and response to the AAV therapy, with a completion date set in 2030 (ClinicalTrials.Gov; NCT03861273). Positive results published in studies and trials emphasize the promise of AAV-based gene therapy for treatment of hemophilia. This success also suggests establishing a wider applicability of AAV therapy as treatment.

There are various other chronic indications where AAV gene therapy could prove advantageous. One such example is Wilson’s Disease. Wilson’s Disease is a rare genetic disease caused by an inherited mutation in the ATP7B gene, which encodes for a copper transporting ATPase mainly expressed in hepatocytes [73,74]. Mutations affecting ATPase cause an accumulation of copper in the body [73,74]. Abnormally high levels of copper can cause damage to organs if left untreated, making it of high priority to regulate these levels [73,74]. Current treatment for Wilson’s Disease is the prescription of chelating agents to manage and remove excess copper from the blood [73,74]. Lifelong treatment is necessary to prevent additional copper buildup [73,74]. Severity of the disease combined with the frequent dosing to manage symptoms suggest Wilson’s Disease would be an attractive candidate for AAV therapies. Indeed, AAV therapies that deliver a functional form of the ATP7B gene to the liver have already been tested in mouse models and were successful in correcting the disease [75,76,77]. Success in mouse models shifted focus to translate these accomplishments in the clinic. Vivet Therapeutics has begun a phase 1/2 trial (ClinicalTrials.Gov; NCT04537377) to observe the safety profile of its AAV-based gene therapy VTX-801, of serotype AAV-Anc80 [78], for Wilson’s Disease. The study aims to deliver functional variants of the ATP7B gene to the liver, and the therapy has already proven efficacious in mice [77]. This clinical trial is still currently recruiting and aims to be completed by the first quarter of 2029.

Gaucher Disease is another chronic indication well-suited to AAV-based gene therapy. Gaucher disease is an autosomal recessive disorder caused by a mutation in the GBA gene [79,80,81], resulting in a lack of the enzyme glucocerebrosidase (GCase) [81]. This enzyme is required to break down lipids in the body. GCase enzyme deficiency results in accumulation of fat in organs such as the spleen and liver [81], leading to a myriad of symptoms including enlarged organs, easy bruising, fatigue, and other potentially more serious neurological effects [81,82]. Currently, more conventional treatment options include enzyme replacement therapy (ERT), which is typically administered every 2 weeks via IV infusions [83]. Additionally, substrate reduction therapy (SRT) may be used in specific patient populations, also requiring daily oral pills [83]. Regardless of treatment, common therapies prescribed to manage symptoms of Gaucher disease are lifelong and demand frequent dosing [84]. Freeline Therapeutics is trying to address the inefficiencies of traditional treatment with its AAV therapy FLT201 (also known as GALILEO-1), which is of AAVS3 serotype [46] and designed to deliver a functional GBA transgene. This therapy is currently at the tail end of a phase 1 clinical trial to assess safety, tolerability, and efficacy. The trial is planned to conclude by the end of 2024 (ClinicalTrialsGov; NCT05324943). 

It is quite clear that AAV therapies have the potential to offer improved treatments of many chronic diseases. With multiple AAV-based gene therapies in various stages in the clinic (ClinicalTrialsGov; NCT04669535, ClinicalTrialsGov; NCT05603312, ClinicalTrialsGov; NCT05568719, ClinicalTrialsGov; NCT00229736), we will likely continue to see the expansion of this approach as groups pursue it to address additional chronic indications. However, genetic cargo design restrictions and immunogenicity associated with AAV are obstacles that persist, emphasizing aspects of this therapy that require further optimization. AAV-based therapy may provide efficacious treatment for a number of monogenic diseases that require a single gene replacement. However, it may not be suitable for every chronic condition, particularly those with a multifactorial etiology.

## 3. Use of pDNA in Gene Therapy

Delivery of naked DNA, such as pDNA, as a therapeutic has gained attention in recent years because of advantages that it offers, especially when compared to other therapies [11,85]. pDNA has demonstrated a strong safety profile in multiple clinical trials [86,87,88,89], confirming high levels of tolerability and minimal toxicity following administration [89]. While AAV therapy has shown cases of genotoxicity as well as unintended integration into the genome across many species [90,91,92,93], these problems have not been reported when delivering pDNA [94,95,96]. Indeed, studies have shown that the risk of integration following delivery of pDNA is negligible [97,98,99]. Unlike the case for AAV, the lack of immune responses directed against pDNA permits repeat dosing [42,43]. The ability to administer this therapy more than once, without the risk of inducing immunity to the therapy and compromising expression, makes pDNA a safe and powerful option for chronic indications where multiple treatments may be necessary.

In addition to its inherent safety characteristics, another advantage of using pDNA is its flexibility when it comes to vector design. Whereas AAV therapies have more strict sequence length limitations [28,29], pDNA has a larger capacity and is better positioned to deliver longer transgenes [100], enabling pDNA-based treatments to target a larger pool of indications [100]. This greater capacity can also lend itself to inclusion of various factors that may help further increase transgene expression [11]. For example, tissue-specific promoters have been shown to help increase efficiency and specificity of treatment by selectively expressing DNA cargo in cells where the specific promoter is active [101]. These, as well as various other modifications, are relatively easy to incorporate into pDNA sequences. 

**Figure 1:**
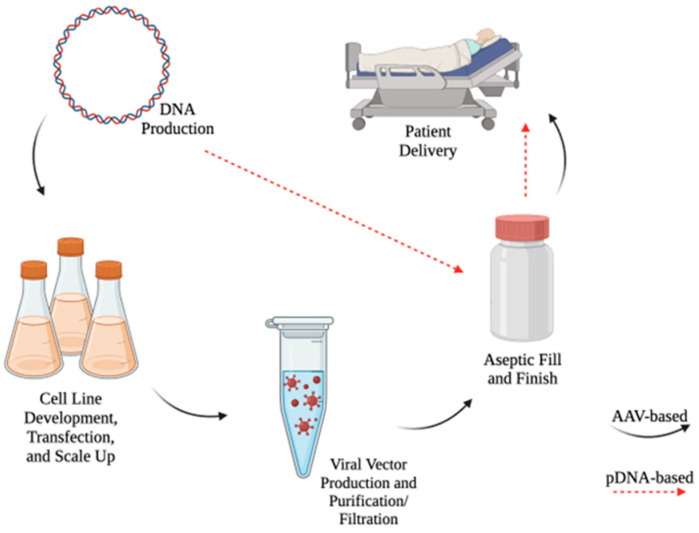
Manufacturing of DNA-based therapies. Large-scale production of AAV-based therapies (black arrows) requires several steps, many of which need optimization, before the product is ready to be administered to the patient. These steps demand a great amount of resources, making the process very expensive, cumbersome, and time consuming. Conversely, producing pDNA (indicated by red dotted lines) on a large scale involves fewer processes, making it faster and more cost-effective. Created with https://BioRender.com (accessed on 13 March 2024).

Aside from the efficacy, safety, and design of gene therapy, a critical consideration needs to be given to the manufacturing and stability of the therapeutic. Large-scale production of AAV is difficult, costly, and time consuming [102,103]. On the other hand, the manufacturing of pDNA avoids most of these disadvantages (Figure 1). The process is relatively inexpensive, scalable, and quick [11]. Additionally, pDNA is more stable than AAV at a wider range of temperatures [104,105]; thus, pDNA therapies do not suffer from some of the cold-chain requirements and stability concerns that apply to AAV delivery systems. 

While pDNA delivery as a method of DNA-based therapy offers several benefits, it is not without limitations. The presence of backbone elements required for large-scale production, such as antibiotic resistance genes or other bacteria-derived sequences, might have negative effects on the patient as well as on expression of the encoded transgene [100,106,107,108]. These can, however, be addressed by incorporating technologies such as minicircle DNA, or fully synthetic linear DNAs, that eliminate bacterial elements and encode only the transgene expression cassette [109,110,111,112].

Although pDNA presents several benefits, direct injection of pDNA does not inherently lead to high and sustained levels of gene expression [113,114]. Therefore, pDNA-based therapy requires an alternative method of delivery to facilitate cellular uptake as well as increase stability and durability of transgene expression.

## 4. Electroporation as a Method for pDNA Delivery

### 4.1. Characteristics of Electroporation

To address the need for high and durable transgene expression levels, different delivery techniques have been developed, including liposomes, particle-mediated gene transfer (‘gene gun’), and EP [115,116,117]. While there has been a lot of excitement surrounding liposomes, nanoparticles, and their derivatives, when delivering nucleic acids, most of the literature focuses on use of these systems for delivery of RNA as opposed to pDNA [118,119]; studies connecting nanoparticles for delivery of pDNA are scarce [120], and most are still in early stages. By comparison, there is an extensive literature outlining EP as a technique previously used in animals, making it an attractive approach for delivery of pDNA in humans [14,121,122].

EP can be used to deliver pDNA to various target tissues. Intradermal EP is often used for DNA vaccines [123], and direct, intratumoral EP is occasionally used in oncology [124,125,126], but the most common target for delivery of the pDNA is skeletal muscle. Injection of pDNA in the muscle is accompanied by short electrical pulses, thereby creating an electrical field which leads to an accumulation of charge across the cell membrane [127]. Past a certain threshold, transient pore formation occurs. Smaller pores stabilize and can increase in size to allow larger molecules, such as pDNA, to enter the cell [127]. Under the right electrical conditions, this permeabilization is temporary and reversible, and after the electrical pulses are complete, the membrane seals within minutes [128,129,130]. By creating pores to facilitate movement of DNA, EP significantly increases DNA uptake by cells, therefore increasing the total expression yield when compared to injections without EP [16,123,131]. By injecting intramuscularly, pDNA/EP converts the muscle cells into therapeutic-producing cells. After intramuscular injection and cell electroporation, the expressed transgene (therapeutic protein) is secreted by the muscle cells and taken up into systemic circulation. Thus, delivery of the protein therapeutic becomes systemic. Although EP of pDNA has been beneficial in boosting transgene expression in smaller species, challenges persist regarding its scalability for human use. Optimized pDNA/EP protocols that are able to achieve robust levels of expression in mice do not always reflect what is achievable in larger animals [132,133]. This lack of translation is due to inherent differences in species anatomy and a much higher dilutional effect in larger animals. Compared to humans, the systemic circulation in mice is confined to lesser volumes; the blood volume in which the expressed transgene can be diluted once entering circulation is 3000-4000-fold smaller in mice than in humans [132,133]. While numerous studies have been conducted to develop more optimized protocols of pDNA/EP in larger species [132,134,135], further research is required to continue bridging the gap between delivery in smaller animals and humans.

### 4.2. Approaching Chronic Indications Using pDNA/EP

**Figure 2:**
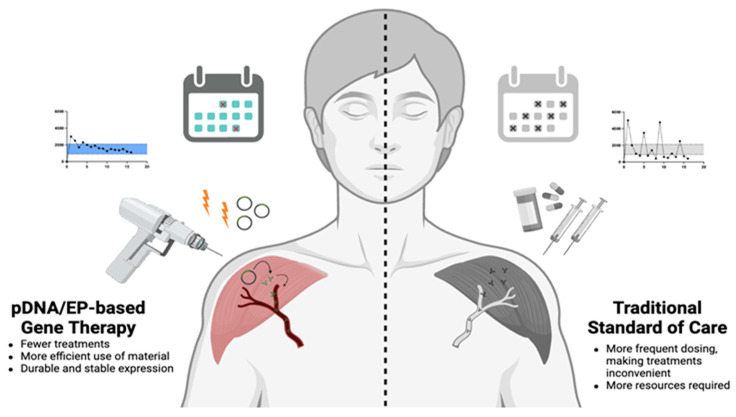
Benefits of pDNA/EP therapy compared to traditional standard of care. pDNA/EP yields robust and stable expression of DNA-encoded transgenes, allowing for fewer treatments than the current standard of care with drugs/biologics delivered via injection or orally. Continuous dosing regimens require more resources to meet the demands of the therapy. Created with https://BioRender.com (accessed on 13 March 2024).

Studies, predominantly in the DNA vaccine field, have demonstrated that in addition to EP being safe and well tolerated, it is also effective in increasing stability and durability of pDNA transgene expression [123,136,137,138]. Such expression characteristics are ideal when evaluating treatments for chronic diseases, as higher and durable expression profiles allow for fewer treatment interventions while remaining efficacious (Figure 2).

While AAV therapies have made significant progress in hemophilia treatment, the issues of immunogenicity persist [69]. Such concerns have not been reported with pDNA-based gene therapies, suggesting pDNA/EP could be a more favorable approach for hemophilia treatments. Studies in immunocompetent mice investigating pDNA/EP of plasmids encoding human FIX (for treatment of hemophilia B) showed high plasma levels of the encoded protein lasting for at least 2 months [139]. Studies have shown similar techniques of pDNA/EP with plasmids encoding FIX in larger animals, such as dogs, suggesting this gene therapy is scalable [140]. Promising results across multiple animal models indicate the benefits of this alternative approach and suggest the therapy can be translated to humans suffering from this indication. 

The short half-life of clotting factors (FVIII and FIX) dictates frequent administration when delivered as recombinant proteins. This limitation can be overcome with pDNA/EP gene therapy by enabling continuous production and release of these factors into circulation. This characteristic of pDNA/EP-based therapy could be beneficial to another blood disorder called chronic neutropenia. Chronic neutropenia is a condition where one exhibits low levels of neutrophils in the blood over an extended period of time [141,142]. Severe chronic neutropenia is treated with recombinant granulocyte colony stimulating factor (G-CSF), which is very efficacious in almost all cases [141,142,143]. However, the short half-life of G-CSF of about 3.5–3.8 h in serum means that the current standard of care often requires daily injections, an approach that is both costly and inconvenient for patients [144,145]. To address this, many attempts have been made to extend the half-life of G-CSF [145,146], and while moderately successful, these improvements do not considerably change the treatment regimen. EP of pDNA presents a potential alternative approach to address these challenges. With studies showing robust and durable expression of genes encoded by pDNA through pDNA/EP [123], there is an opportunity to concurrently increase expression levels as well as improve the pharmacokinetic profile of G-CSF, allowing for significantly less frequent dosing. 

Hormone deficiency disorders, such as Addison’s disease, represent other chronic conditions that are driven by lack of a protein factor in circulation. Addison’s disease is characterized by insufficient hormonal production by the adrenal gland [147,148]. Current treatments for Addison’s disease are lifelong, consisting of daily oral pills taken to replace the missing hormones [147,148]. Studies in pigs showed that plasmids encoding growth hormone-releasing hormone (GHRH) delivered using pDNA/EP [149] stimulated growth hormone (GH) secretion over at least 2 months with a single treatment. Applying pDNA/EP to the treatment of Addison’s disease could potentially produce comparable outcomes, reflecting the success seen in pigs and thus reducing frequency of treatments necessary to manage the disease. While this study highlights the potential translation of a novel approach to treat Addison’s disease, it also demonstrates the versatility of pDNA/EP, emphasizing its ability to deliver diverse payloads. 

In line with the adaptability of DNA-based medicines, pDNA/EP can also enhance the delivery of monoclonal antibodies for treating chronic diseases [15]. Multiple in vivo studies have demonstrated effective and durable delivery of DNA-based monoclonal antibodies using pDNA/EP [150,151,152], and phase 1 clinical trials are already in progress to evaluate the safety profile of this approach [151] (ClinicalTrialsGov; NCT05293249). 

Chronic viral infections, a subset of chronic diseases, like those caused by the human immunodeficiency virus (HIV), could benefit from DNA-based methods for delivering antibodies. When left untreated, HIV leads to acquired immunodeficiency syndrome (AIDS) [153,154]. Although substantial progress has been made in developing treatments for HIV in recent decades, allowing people who have been diagnosed to live relatively normal lives, treatments are continuous and administered frequently [153,154]. Most antiretroviral therapies (ART) require daily treatment, comprised of one or more drugs [153,154] that potently decrease the viral load [154,155]. While treatment helps resolve symptoms and decreases chances of transmission, it does not cure HIV [154,155,156]. As a result, treatment needs to be maintained for life, making the dosing regimen of daily pills even more strenuous and time consuming. For patients where daily oral ART has accomplished viral suppression, treatment regimens can be switched to longer-acting injectable therapy [156,157]. This longer-acting therapy is administered once every 2 months and does not need to be taken with any other HIV medications [156,157]. However, these longer-acting treatments still require relatively frequent administration, making pDNA/EP an attractive alternative approach. Studies in mice and non-human primates (NHP) have demonstrated that DNA-encoded broadly neutralizing antibodies against HIV can be efficiently delivered with EP [158]. The DNA-encoded antibodies delivered by EP not only showed stable expression in mice for almost a year, but were also efficacious in NHPs against multiple HIV strains, highlighting potential advantages of pDNA/EP as an approach for treatment and possible prevention of this chronic disease [158]. 

Similarly, chronic inflammatory conditions represent a large disease area that could benefit from pDNA/EP treatment. For example, plaque psoriasis is a chronic, relapsing, immune inflammatory disorder, characterized by patches of dry, itchy, raised skin covered with a whitish buildup of dead cells [159,160]. Many biologics used to manage symptoms of plaque psoriasis are monoclonal antibodies. Dosing frequencies range from 4 to 8 weeks on average and require lifelong administration to remain efficacious [160,161]. These biologics could be delivered with pDNA/EP, potentially just once or twice a year, facilitating a durable and long-lasting expression of encoded antibodies to greatly improve the dosing regimen.

## 5. Concluding Remarks

In recent years, substantial progress has been made in the domain of DNA-based therapy, confirming its capacity to act as an alternative to conventional treatments [1]. AAV-based therapy has come a long way, with several therapies already FDA-approved, and many others being currently evaluated in clinical trials [18]. However, limitations of AAV remain, including complications due to immunity and large-scale manufacturing difficulties [42,103]. Continued work towards developing methods that avoid these obstacles is critical. Doing so will help to increase the therapeutic impact of AAV-based gene therapy and is of utmost importance in addressing chronic indications where multiple rounds of AAV might be necessary. 

pDNA-based therapy is another DNA-based approach that has gained attention. pDNA demonstrates a strong safety profile and has low immunogenicity [11,151]. However, injection of pDNA on its own is incapable of producing expression levels that are high and stable enough to treat many indications [113,114]. Implementation of EP as a delivery method has helped to abrogate these drawbacks, enabling high, robust, and durable expression of pDNA-encoded therapeutics in vivo across many species [14,121,132]. Durable expression profiles support the use of this approach to address many chronic indications. In multiple disease models, pDNA/EP-based treatments have shown success, yielding robust expression and requiring fewer interventions to maintain therapeutic levels [139,149,158]. While success across various species suggests translatability to humans, scalability of pDNA/EP treatments requires further investigation to ensure efficient production of the biologic at therapeutic levels [132,133,134].

The pDNA/EP approach to gene therapy has proven its versatility, with the ability to deliver a diverse range of cargo. With previous success comes the hope that this approach will improve the landscape of chronic disease treatments, with one potential avenue being Glucagon-like peptide-1 (GLP-1) receptor agonists currently used to treat type 2 diabetes and obesity [162,163,164]. The current treatment necessitates weekly administration to maintain therapeutic efficacy, highlighting the potential of pDNA/EP as an appealing approach to revamp the dosing schedule for these prevalent chronic conditions. [162,163,164]. 

With approaches being investigated to address current limitations, an as new data emerge encouraging expansion into new indications, we can feel confident that DNA-based therapies will continue to flourish.

## Figures and Tables

**Table 1:** Comparison of pDNA/EP and AAV DNA-based therapies. Characteristics described highlight a list of important factors that should be considered when contemplating which therapy is most suitable for a given indication. The number of asterisks indicates the strength of a given characteristic (arbitrary scale).

Feature	pDNA/EP Gene Therapy	AAV Gene Therapy
Durability	Months to years	Months to many years
Clinical safety of technology	***	*
Redosable	****	*
Large genetic payload	****	**
Opportunity	Systemic activity, Genetic disease, Chronic disease	Specific tissue target, Genetic disease, Chronic disease
Large scale manufacturing	****	*
Freedom from cold chain	****	*

**Table 2:** Tissue tropism for 13 natural AAV serotypes in non-human primates and humans.

Natural AAV Serotype Tissue Tropisms	
Tissue Type	AAV Serotype
Skeletal Muscle	AAV1, AAV2, AAV6, AAV7, AAV8, AAV9, AAV12
Cardio-myocytes	AAV1, AAV4, AAV6, AAV7, AAV8, AAV9
Endothelial Vascular Smooth Muscles	AAV1, AAV5, AAV7
Inner Ear Cells	AAV3
Retinal Cells	AAV1, AAV2, AAV4, AAV5, AAV7, AAV8, AAV9, AAV10
CNS	AAV1, AAV2, AAV4, AAV5, AAV7, AAV9, AAV10, AAV11
Airway Epithelia	AAV4, AAV5, AAV6, AAV9, AAV10, AAV12
Hepatocyes	AAV2, AAV3, AAV5, AAV7, AAV8, AAV9, AAV10, AAV11
Salivary Glands	AAV12
Pancreatic Cells	AAV8, AAV9, AAV10
Small Intestine Cells	AAV10, AAV11
Colon Cells	AAV10
Lymph Nodes	AAV10, AAV11
Leydig Cells	AAV9
Adrenal Glands	AAV10, AAV11
Renal Tissue	AAV2, AAV4, AAV8, AAV9, AAV10, AAV11
